# Real-World Effectiveness of Nirsevimab Against Respiratory Syncytial Virus: A Test-Negative Case-Control Study

**DOI:** 10.1101/2024.09.12.24313545

**Published:** 2024-09-12

**Authors:** Hanmeng Xu, Camilla Aparicio, Aanchal Wats, Barbara L. Araujo, Virginia E. Pitzer, Joshua L. Warren, Eugene D. Shapiro, Linda M. Niccolai, Daniel M. Weinberger, Carlos R. Oliveira

**Affiliations:** 1 Department of Epidemiology of Microbial Diseases, Yale School of Public Health, New Haven, CT, USA; 2 Department of Pediatrics, Section of Infectious Diseases and Global Health, Yale School of Medicine, New Haven, CT, USA; 3 Department of Biostatistics, Yale School of Public Health, New Haven, CT, USA; 4 Department of Biostatistics, Section of Health Informatics, Yale School of Public Health, New Haven, CT, USA; 5 Department of Biomedical Informatics and Data Science, Yale School of Medicine, New Haven, CT, USA

## Abstract

**IMPORTANCE:**

Nirsevimab, a long-acting monoclonal antibody, has demonstrated efficacy against RSV-related lower respiratory tract infections (LRTIs) in clinical trials. Post-licensure monitoring is essential to confirm these benefits in real-world settings.

**OBJECTIVE:**

To evaluate the real-world effectiveness of nirsevimab against medically attended RSV infections in infants and to assess how effectiveness varies by disease severity, dosage, and time since immunization.

**DESIGN, SETTING, AND PARTICIPANTS:**

This test-negative case-control study used inpatient, outpatient, and emergency room data from the Yale New Haven Health System. Nirsevimab-eligible infants who were tested for RSV using polymerase chain reaction between October 1, 2023 and May 9, 2024 were included. Cases were infants with confirmed RSV infections; controls were those who tested negative.

**EXPOSURE:**

Nirsevimab immunization, verified through state immunization registries.

**MAIN OUTCOMES AND MEASURES:**

Effectiveness was estimated using multivariable logistic regression, adjusting for age, calendar month, and individual risk factors. Separate models examined effectiveness by clinical setting, disease severity, dose, and time since immunization. Broader outcomes, including all-cause LRTI and LRTI-related hospitalization, were also analyzed, with stratification by early and late respiratory seasons.

**RESULTS:**

The analytic sample included 3,090 infants (median age 6.7 months, IQR 3.6–9.7), with 680 (22.0%) RSV-positive and 2,410 (78.0%) RSV-negative. 21 (3.1%) RSV-positive and 309 (12.8%) RSV-negative infants received nirsevimab. Effectiveness against RSV infection was 68.4% (95% CI, 50.3%–80.8%). Effectiveness was 61.6% (95% CI, 35.6%–78.6%) for outpatient visits and 80.5% (95% CI, 52.0%–93.5%) for hospitalizations. The highest effectiveness, 84.6% (95% CI, 58.7%–95.6%), was observed against severe RSV outcomes requiring ICU admission or high-flow oxygen. Although effectiveness against RSV infections declined over time, it remained significant at 55% (95% credible interval, 16%–75%) at 14 weeks post-immunization. Protective effectiveness was also observed against all-cause LRTI and LRTI-related hospitalizations during peak RSV season (49.4% [95% CI, 10.7%–72.9%] and 79.1% [95% CI, 27.6%–94.9%], respectively). However, from February to May, when RSV positivity was low, effectiveness against these broader outcomes was negligible.

**CONCLUSIONS AND RELEVANCE:**

Nirsevimab provided substantial protection against RSV-related outcomes for at least three months. These findings support the continued use of nirsevimab and provide evidence that may help build public confidence in the immunization program.

## Introduction

Respiratory syncytial virus (RSV) is a major cause of acute lower respiratory tract infection (LRTI), particularly affecting newborns and infants under 1 year of age. Globally, RSV is responsible for approximately 1.4 million hospital admissions and 13,300 in-hospital deaths annually among infants aged 0–6 months [1]. The recent introduction of prophylactic interventions, such as nirsevimab, provides a promising strategy to mitigate RSV’s impact on this vulnerable population.

Nirsevimab, a long-acting monoclonal antibody, was licensed by the United States (US) Food and Drug Administration in July 2023 after demonstrating safety and efficacy in prelicensure trials[2]. These trials reported 79% efficacy (95% confidence interval [CI], 69–86) against medically attended RSV, 81% (95% CI, 62–90) against RSV requiring hospitalization, and 90% (95% CI, 16–99) against severe RSV requiring intensive care unit (ICU) admission[3]. Following its licensure, the Centers for Disease Control and Prevention’s (CDC) Advisory Committee on Immunization Practices (ACIP) recommended nirsevimab for infants under 8 months entering their first RSV season and for high-risk infants aged 8–19 months[4].

While pre-licensure clinical trials demonstrated strong efficacy, it is essential to validate these findings through post-licensure studies that assess nirsevimab’s effectiveness in real-world settings. Such studies are needed to ensure that the protective effects of immunizations remain as they are being used in routine clinical practice, where factors like comorbidities, access to care, and clinician practices can influence outcomes. Early real-world data from the 2023–2024 RSV season in Europe and the US show effectiveness ranging from 70% to 90% against hospitalization for RSV-associated LRTI[5–9]. However, several gaps in knowledge remain. Specifically, there are limited data on nirsevimab’s long-term effectiveness, its impact at different dosages, and its ability to prevent milder RSV cases. Furthermore, there is a need for further exploration of its effectiveness in diverse populations, particularly those with underlying health conditions. To address these gaps, this study aims to evaluate nirsevimab’s real-world effectiveness in a diverse US patient population and examine how protection varies over time, by disease severity, and by dosage used.

## Methods

### Study Design and Study Population

The effectiveness of nirsevimab against medically attended RSV infection was estimated using the test-negative case-control study design. The study population included all patients who were born after October 1st, 2022, were tested for RSV due to a suspected acute respiratory infection (ARI)[10], and received care in a facility affiliated with the Yale New Haven Health System (YNHHS) between October 1^st^ 2023 and May 9^th^ 2024. The YNHHS is the largest health system in Connecticut, and consists of five integrated hospital networks, 30 emergency or urgent care centers, and over 130 outpatient clinics from Westchester County, New York, to Rhode Island, all integrated using a single electronic health record (EHR) system.

**Figure 1.**
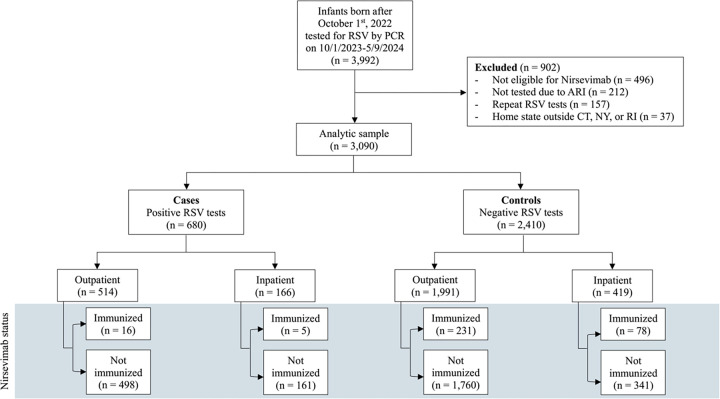
Selection of RSV test records Abbreviations: RSV, respiratory syncytial virus; PCR, polymerase chain reaction; ARI, acute respiratory infection; CT, Connecticut; NY, New York; RI, Rhode Island.

Patients were excluded from these analyses if they were not age-eligible for nirsevimab when it became available on October 1^st^ 2023[11], or if they resided outside Connecticut, New York, or Rhode Island. The geographic restriction was implemented to ensure that immunization records could be verified through state immunization registries, which are directly integrated with the YNHHS EHR. Infants were considered eligible for nirsevimab if they were born during the season (after October 1st, 2023), or if they were under 8 months old and entering their first RSV season, or if they were between 8 and 12 months old with a risk factor for severe RSV (see Figure 1 for the detailed inclusion process and Table S1 for the definitions for risk factors).

### Data Sources and Study Definitions

For all the infants who met the eligibility criteria, reviews of medical records and state immunization registry searches were conducted to capture information on patient characteristics, immunization history, and potential confounders. Relevant clinical and laboratory data associated with each patient’s RSV test, such as chief complaints, problem lists, encounter diagnoses, and presence of any other acute or chronic diseases were abstracted by trained investigators from the EHR. The clinical outcomes following hospitalization were also recorded, such as hospital and ICU length of stay, and maximum respiratory support needed during hospitalization. Patient characteristics including age, race and ethnicity, gestational age, birth weight, and type of insurance were also abstracted.

Cases were defined as infants with a medically attended RSV infection confirmed by nasopharyngeal polymerase chain reaction (PCR). Controls were infants with ARI who tested negative for RSV. For a given patient, if there was more than one positive test during the study period, only the first was included in the study. If an eligible control had more than one negative test within 14 days, the first negative test was selected for this period. The primary exposure of interest in our study was the nirsevimab immunization status. Only documented immunization dates were included in the analysis. Infants were classified as “immunized” if they received a dose of nirsevimab prior to their RSV test.

### Statistical analysis

The characteristics of the study population were summarized using frequency distributions and measures of central tendency. Univariate analyses were performed to compare RSV-positive patients with negative controls, and unimmunized with immunized infants. Covariate balance between groups was assessed to detect potential confounders using standardized mean differences (SMD), with absolute SMDs of less than 0.20 indicating adequate balance.

For our primary analysis, the effectiveness of nirsevimab against medically attended RSV infection was quantified using all eligible patients in our study population. Effectiveness was calculated as one minus the odds ratio (OR) of immunization with nirsevimab among cases and controls, using logistic regression. Adjusted effectiveness estimates were derived using multivariable models that controlled for age, calendar month, and the presence of at least one risk factor. Non-collinear potential confounders were selected for the final models through backward selection based on the Akaike information criterion (AIC). Model formulas and AIC values are provided in the supplementary materials (Figure S1, Table S2).

For our secondary aims, separate models were fitted to analyze the data based on clinical setting (inpatient vs outpatient), disease severity, nirsevimab dosage used, and time from immunization. For the severity analysis, patients were considered to have severe disease if they were hospitalized within 14 days of RSV testing and required either transfer to a pediatric ICU or high levels of respiratory support during hospitalization, such as high-flow nasal cannula (≥2 liters per minute), continuous or bilevel positive airway pressure, or invasive mechanical ventilation. The extent to which the effectiveness of nirsevimab decreased over time was quantified using logistic regression within a Bayesian framework. The parameter representing the effectiveness of immunization was time-varying at bi-weekly intervals of time since immunization. These models used weakly-informative prior distributions and imposed a monotonic structure on the regression coefficients that represent nirsevimab’s effectiveness for increasing time since immunization. Medians and 95% credible intervals were calculated from the collected posterior samples, and convergence was evaluated using trace plots (Figure S6). A comprehensive description of the Bayesian models is provided in the supplementary material.

The effectiveness of nirsevimab was also assessed using broader endpoints, including all-cause lower respiratory tract infection (LRTI) and all-cause LRTI hospitalization across the entire respiratory season, with additional stratification by early (October to January) and late (February to May) periods.

### Sensitivity analyses

Several sensitivity analyses were conducted to assess the robustness of our findings. First, we assessed for differences in effectiveness when employing different exposure and outcome definitions. Specifically, we explored restricting our analysis to only medical visits where encounter diagnoses indicating lower respiratory tract infection (LRTI) were recorded as either a primary or secondary diagnosis (Table S1). We also explored restricting controls to only those who tested positive for other respiratory viruses (i.e., influenza, adenovirus, rhinovirus, parainfluenza). In terms of exposure, we explored defining patients as “immunized” if they received nirsevimab ≥7 days prior to RSV testing, as was done in earlier reports to account for the mean RSV incubation period and the time required to reach peak antibody concentration[9]. Second, we assessed whether excluding infants whose mothers received the maternal RSV vaccine or those who were born during the previous RSV season would significantly alter the results. Last, we repeated our analysis using the hepatitis B vaccine as a “sham” exposure, as previously described [12, 13]. Since the hepatitis B vaccine is recommended to be given to all newborns but does not affect the risk of RSV infection, we expect that in the absence of bias, the proportions of cases and of controls who were immunized with the hepatitis B vaccine will not be significantly different.

Further details on study definitions and statistical analysis are provided in the Supplemental Methods. All analyses were conducted in R, version 4.3.1[14]. The code for the analysis can be found at https://github.com/Hanmeng-Xu/RSV_mAb_VE.

The institutional review board (IRB) at the Yale University School of Medicine approved the study (HIC:2000036550). The funders of this study had no role in study design; collection, analysis, and interpretation of data; writing the report; and the decision to submit the report for publication.

## Results

### Study population

Between October 1, 2023, and May 9, 2024, a total of 3,992 RSV tests were performed within the YNHH system on infants born after October 1, 2023. Of these, 3,090 met our eligibility criteria and were included in the analysis (Figure 1). The analytic sample consisted of 680 RSV-positive and 2410 RSV-negative patients. Most RSV tests occurred during emergency department or urgent care clinic visits (n= 2,505, 81.1%) in the months of December and January (Figure S2). The median age at the time of RSV testing was 6.7 months (IQR 3.6 to 9.7 months). RSV-positive cases were slightly younger than RSV-negative controls (6.1 vs. 6.9 months, p = 0.007) and had a lower proportion of prematurity (11.2% vs. 14.2%, p = 0.045). However, other demographic and clinical factors, including sex, race/ethnicity, insurance type, and prevalence of comorbidities, were comparable between the two groups (Table 1).

The overall uptake of nirsevimab in the study sample was 10.7% (330/3,090). Among those who received nirsevimab before RSV testing, 71.2% (n = 235) received the 50 mg dose, while 28.8% (n = 95) received the 100 mg dose. Correlates of immunization are detailed in Table S3.

Overall, 24.4% (166/680) of RSV-positive cases resulted in hospitalization. Among those hospitalized, 58.4% (97/166) required more than two liters of respiratory support, and 13.8% (23/166) required admission to the ICU (Table S4).

### Effectiveness of nirsevimab

**Figure 2.**
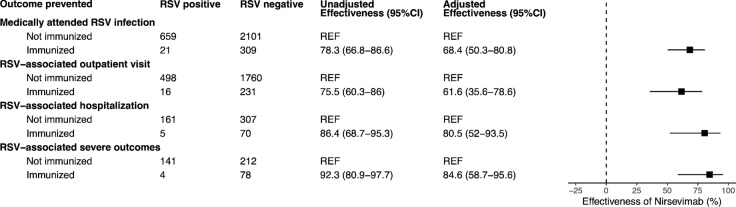
Effectiveness of nirsevimab against medically attended RSV, by clinical setting and severity. Adjusted models controlled for age, calendar month, and the presence of at least one risk factor (see Table S2).

The adjusted effectiveness of nirsevimab against any medically attended RSV infection was 68.4% (95% CI: 50.3–80.8%). Effectiveness was 61.6% (95% CI: 35.6–78.6%) for preventing RSV-associated outpatient visits, 80.5% (95% CI: 52.0–93.5%) for preventing hospital admissions, and 84.6% (95% CI: 58.7–95.6%) for preventing severe RSV (Figure 2). These findings aligned with existing clinical trials and observational data (Figure S3). The effectiveness estimates for the 100 mg and 50 mg doses were comparable, with overlapping confidence intervals (Figure S4).

**Figure 3.**
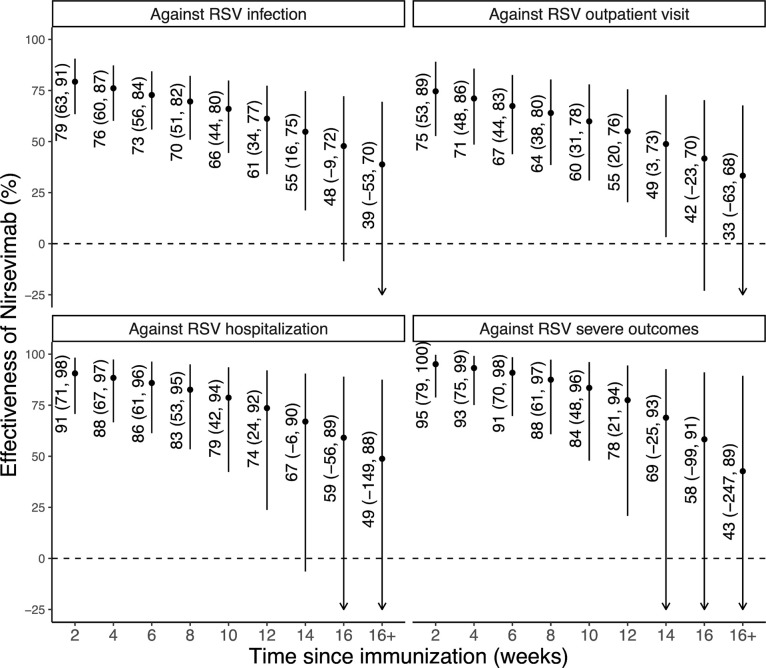
Effectiveness of nirsevimab by time since immunization. The dots represent the median estimates of effectiveness of nirsevimab in preventing the various clinical outcomes, and the error bars indicate the 95% credible intervals of the estimates.

Nirsevimab effectiveness against medically attended RSV infection decreased from 79% at 2 weeks post-immunization to 55% at 14 weeks post-immunization. A similar pattern of waning effectiveness was observed across different outcomes (Figure 3); our results were comparable to those derived from clinical trial data (Figure S5).

**Figure 4.**
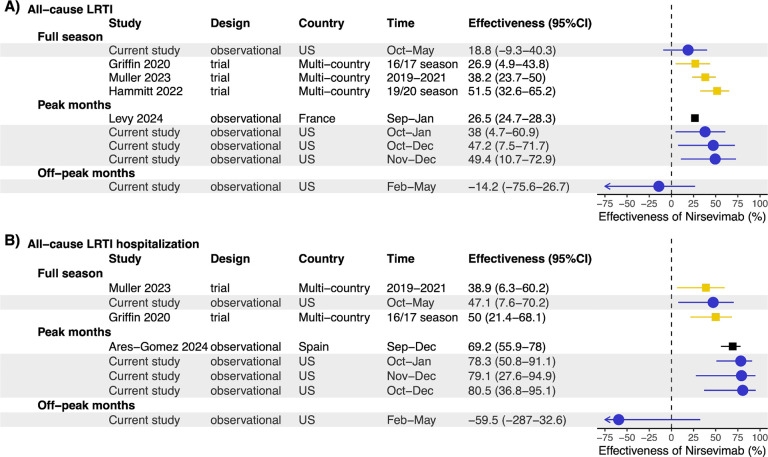
Forest plot of nirsevimab effectiveness against (A) all-cause LRTI and (B) all-cause LRTI hospitalization, stratified by time. Current study estimates are shown in blue. For comparison, estimates from three previous studies are also included. Pre-licensure clinical trial estimates are shown in gold, and other post-licensure study estimates are in black. Estimates were stratified by time (full season[30, 34, 35], peak months[5, 21], off-peak months) Only the estimate for age group 3–12 months was shown for Levy et al. 2024[21].

Protective effectiveness was observed against all-cause LRTI and all-cause LRTI hospitalization during the peak months of the RSV season (49.4% [95% CI: 10.7–72.9%] and 79.1% [95% CI: 27.6–94.9%] during November and December, respectively), comparable to results from other studies (Figure 4). Given that RSV was the predominant virus during these months (RSV positivity rate 39.3%), the above estimate largely reflects the effectiveness of nirsevimab against RSV. In contrast, between February and May, when the RSV positivity dropped below 3.9%, there was no effectiveness of nirsevimab against all-cause LRTI and all-cause LRTI hospitalizations (Figure 4).

All sensitivity analyses generated consistent results, with less than 10% differences in point estimates of effectiveness (Table S5). As expected, the proportions of cases and controls that received the hepatitis B vaccine were nearly identical (54% and 57%, respectively, p=0.18), and the corresponding effectiveness of the hepatitis B vaccine against RSV was not statistically significant (Table S5).

## Discussion

In this study, we provide robust evidence supporting the real-world benefits of nirsevimab, with an adjusted effectiveness of 68% against medically attended RSV infections. Our data indicates that effectiveness was higher for RSV-related hospitalizations (80.5%) and severe RSV disease (84.6%). These findings align with the pre-licensure clinical trials, which reported 77–83% efficacy against RSV-related hospitalizations[15]. Emerging evidence from post-licensure studies, including a recent meta-analysis that estimated the effectiveness of 88.4% (95% CI: 84.7–91.2%) against RSV-related hospitalizations[16], further supports the findings of this study.

Our study makes several important contributions to the existing literature. First, we measure the protective effect of nirsevimab in a diverse US patient population where historically minoritized racial and ethnic groups make up the majority (>50%) of the study sample. This is notable because most previous effectiveness estimates have come from studies conducted primarily in European countries, which have distinct racial and ethnic compositions, different socioeconomic contexts, and considerably higher nirsevimab coverage. For instance, fifteen out of sixteen real-world effectiveness studies to date have been conducted in Western Europe[5–8, 17–27], where nirsevimab coverage in the target population often exceeded 70%.

Second, previous analyses, including the only US-based report [9], have primarily focused on RSV-associated hospitalizations, with limited evaluation of effectiveness against medically attended outpatient visits, which represent a substantial portion of the RSV burden [28, 29]. Our study addresses this gap but also extends the evaluation of nirsevimab to its impact on broader outcomes, such as all-cause LRTI. Prior post-licensure studies reported effectiveness of 69.2% (55.9–78.0%) against all-cause LRTI hospitalizations[5], nearly double the 39% efficacy observed in Phase III trials against these non-specific outcomes[30]. Our findings align more closely with clinical trial data, and, notably, we found no significant protective effect against all-cause LRTI outside the peak RSV season when a negligible proportion of LRTIs were due to RSV.

Third, our study provides valuable insights into the temporal dynamics of nirsevimab’s effectiveness. Given that our study spanned the entire RSV season, we were able to assess how nirsevimab’s effectiveness wanes over time—a factor less emphasized in earlier studies with shorter observation periods. While effectiveness did decline over time, the protective effects remained statistically significant for at least 14 weeks post-immunization. This waning pattern is consistent with what is known about the pharmacokinetics of monoclonal antibodies and the natural decay of passive immunity[31]. The observed decline in effectiveness beyond 14 weeks, though preliminary and based on estimates with wide uncertainty intervals, underscores the importance of timing in administering nirsevimab, especially in regions with prolonged RSV seasons.

Our study has several limitations. First, the prioritization of high-risk infants for immunization during the early roll-out phase may have introduced confounding by indication[32]. However, we adjusted for this potential effect in our analysis and conducted several sensitivity analyses that suggest residual confounding by unmeasured factors is less likely. Second, although our study benefited from a large sample size, the low uptake of nirsevimab resulted in limited statistical power and wide confidence intervals (CIs) for certain comparisons, such as effectiveness by the number of doses received. Third, because few cases were immunized more than 14 weeks before RSV testing, the effectiveness estimates beyond this period have wide credible intervals and should be interpreted with caution. Fourth, under-ascertainment of immunization or prior infections may have biased our results toward the null. However, the uptake of nirsevimab in our study is very similar to that reported by other US-based studies and CDC coverage estimates for Connecticut (7.7% uptake) [9, 33]. Furthermore, our “sham exposure” analysis suggests that our estimate of nirsevimab’s effectiveness was not likely confounded by factors associated with immunization ascertainment.

## Conclusion

This study confirms that nirsevimab is highly effective at preventing RSV-associated outpatient visits, hospitalizations, and severe disease requiring ICU admission or high-flow oxygen. Its effectiveness persisted for at least three months post-vaccination, consistent with results from randomized trials. These findings reinforce the benefits of RSV immunoprophylaxis and support US guidelines recommending nirsevimab for all infants entering their first RSV season.

## Supplementary Material

Supplement 1

## Figures and Tables

**Table 1.** Characteristics of included cases and controls, October 1^st^ 2023 – May 9^th^ 2024.

	Overall, N = 3,090^1^	Cases, N = 680^1^	Controls, N = 2,410^1^	Standardized Mean Difference

**Sex**				0.05
Female	1,317 (42.6%)	279 (41.0%)	1,038 (43.1%)	
Male	1,772 (57.3%)	401 (59.0%)	1,371 (56.9%)	
(Missing)	1 (0.0%)	0 (0.0%)	1 (0.0%)	
**Age at testing (months)**				−0.14
Median (IQR)	6.7 (3.6, 9.7)	6.1 (3.4, 9.2)	6.9 (3.7, 9.9)	
**Race and ethnicity**				0.13
Hispanic	1,328 (43.0%)	280 (41.2%)	1,048 (43.5%)	
White non-Hispanic	820 (26.5%)	201 (29.6%)	619 (25.7%)	
Black non-Hispanic	533 (17.2%)	112 (16.5%)	421 (17.5%)	
Other non-Hispanic^2^	161 (5.2%)	26 (3.8%)	135 (5.6%)	
Unknown	248 (8.0%)	61 (9.0%)	187 (7.8%)	
**Birth weight**				0.10
Median (IQR)	3,214.3 (2,824.9, 3,563.8)	3,265.0 (2,875.0, 3,576.2)	3,194.5 (2,805.1, 3,553.6)	
N missing (% missing)	783 (25.3%)	184 (27.1%)	599 (24.9%)	
**Prematurity (< 37 weeks)**	418 (13.5%)	76 (11.2%)	342 (14.2%)	0.11
**Pulmonary diseases**	156 (5.0%)	26 (3.8%)	130 (5.4%)	0.07
**Cardiac diseases**	152 (4.9%)	30 (4.4%)	122 (5.1%)	0.03
**Anemia**	94 (3.0%)	15 (2.2%)	79 (3.3%)	0.07
**Having at least one risk factor** ^3^	750 (24.3%)	150 (22.1%)	600 (24.9%)	0.07
**Insurance type**				0.09
Private	983 (31.8%)	231 (34.0%)	752 (31.2%)	
Public	2,088 (67.6%)	442 (65.0%)	1,646 (68.3%)	
Uninsured	19 (0.6%)	7 (1.0%)	12 (0.5%)	
**mAb status**				0.37
No	2,760 (89.3%)	659 (96.9%)	2,101 (87.2%)	
Yes, 100mg dose	95 (3.1%)	6 (0.9%)	89 (3.7%)	
Yes, 50mg dose	235 (7.6%)	15 (2.2%)	220 (9.1%)	

1 *n* (%)

2 Inclusing Asian, Pacific Islander, Middle Eastern or Northern American, American Indian or Native American by self-reporting.

3 Have at least one of the following conditions recorded in the infant’s medical history or diagnosis records: 1) Anemia; 2) Immunodeficiency (e.g. transplantation history, leukemia, etc.); 3) Cardiac diseases (including congenital heart diseases diagnosed at birth or any reporting of heart conditions); 4) Pulmonary diseases; 5) Down syndrome; 6) Small for gestational age (birth weight < 2,500 grams); 7) Prematurity (gestational age less than 37 weeks).

## Key Points

### Question:

What is the effectiveness of nirsevimab against medically attended respiratory syncytial virus (RSV) infections in infants?

### Findings:

680 RSV test-positive cases and 2,410 RSV test-negative controls were included in this test-negative case-control study. Nirsevimab’s effectiveness was 69% against RSV infections, 81% against RSV-associated hospitalization, and 85% against severe RSV disease. Effectiveness against RSV infection declined from 79% at 2 weeks post-immunization to 55% at 14 weeks post-immunization.

### Meaning:

Nirsevimab provides strong protection against a wide range of RSV outcomes, but its effectiveness diminishes over time. These data can be utilized to optimize nirsevimab’s implementation and sustain its uptake.
