# Supplementary material for: Real-World Effectiveness of Nirsevimab Against Respiratory Syncytial Virus: A Test-Negative Case-Control Study
Source: medRxiv. 2024 Sep 12:2024.09.12.24313545. Preprint. [Version 1] doi: 10.1101/2024.09.12.24313545 (PMC11419242; doi:10.1101/2024.09.12.24313545)
Supplement: Supplement 1 [file NIHPP2024.09.12.24313545v1-supplement-1.pdf]

medRxiv preprint doi: https://doi.org/10.1101/2024.09.12.24313545; this version posted September 12, 2024. The copyright holder for this preprint (which was not certified by peer review) is the author/funder, who has granted medRxiv a license to display the preprint in perpetuity.

It is made available under a CC-BY 4.0 International license .

Real-World Effectiveness of Nirsevimab Against Respiratory Syncytial Virus: A Test-Negative Case-Control Study

Supplementary Materials

Table of contents

SECTION 1. SUPPLEMENTAL FIGURES REFERENCED IN THE MAIN MANUSCRIPT
Figure S1 Correlation between potential confounders in the test-negative case-control analysis. 1
Figure S2 RSV tests and nirsevimab doses during the study period. 2
Figure S3 Overview of nirsevimab effectiveness: Current study estimates in context with prior research. 3
Figure S4 Effectiveness of nirsevimab against RSV infections by dose, clinical setting, and disease severity. 4
Figure S5 Effectiveness of nirsevimab against RSV-associated LRTI by time since immunization. 5
Figure S6 Trace plots for the coefficients of waning effectiveness. 6
SECTION 2. SUPPLEMENTAL TABLES REFERENCED IN THE MAIN MANUSCRIPT
Table S1. Definition of key clinical outcomes and risk factors. 7
Table S2. Variable selection for the multivariable logistic regression models. 8
Table S3. Comparison of immunized and unimmunized patients. 9
Table S4. Clinical characteristics of RSV-positive cases. 10
Table S5. Sensitivity Analysis. 11
SECTION 3. SUPPLEMENTAL METHODS
Estimating the effectiveness of nirsevimab over time 12
STROBE Statement—Checklist of items that should be included in reports of case-control studies 14
SECTION 4. SUPPLEMENTAL REFERENCES 16

medRxiv preprint doi: https://doi.org/10.1101/2024.09.12.24313545; this version posted September 12, 2024. The copyright holder for this preprint (which was not certified by peer review) is the author/funder, who has granted medRxiv a license to display the preprint in perpetuity.

It is made available under a CC-BY 4.0 International license .

SECTION 1. SUPPLEMENTAL FIGURES REFERENCED IN THE MAIN MANUSCRIPT

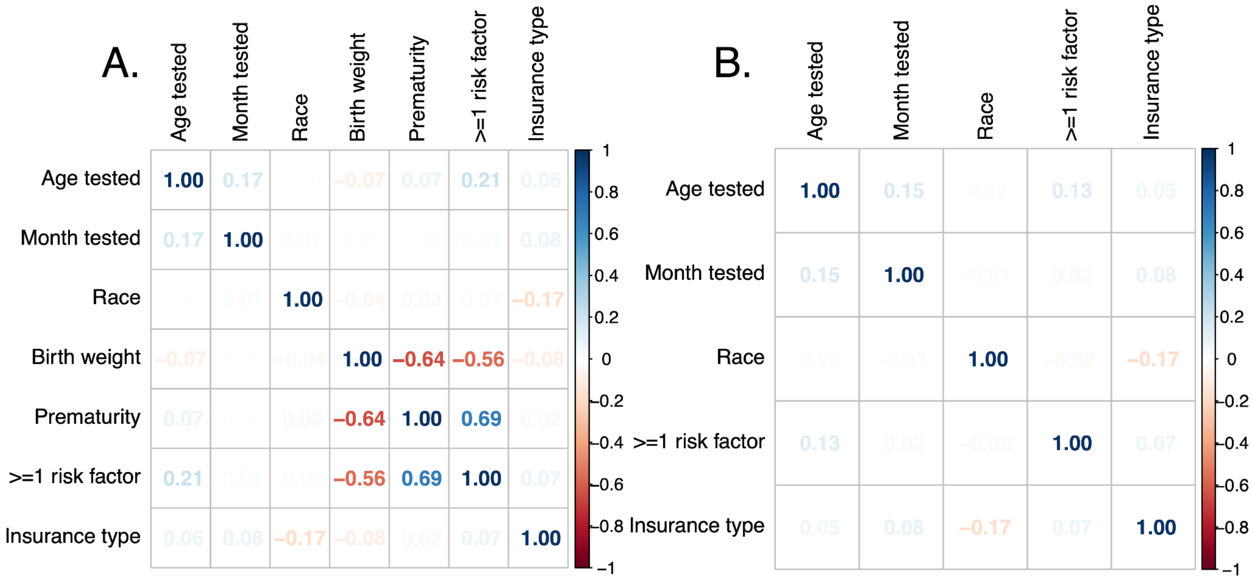

Figure S1. Correlation between potential confounders in the test-negative case-control analysis. Multivariate logistic regression was used to estimate nirsevimab effectiveness against various clinical outcomes. Potential confounders were selected using backward selection from variables in the initial model (panel A), including age at testing (<3, 3-5, 6-8, 9-11, ≥12 months), calendar month of testing, race/ethnicity, birth weight, prematurity (gestational age <37 weeks), presence of at least one risk factor (see Table S1), and insurance type (private, public, uninsured). The numbers show the correlation coefficients between any of the two variables, and a value larger than 0.5 was defined as a moderate or strong correlation. Due to collinearity between low birth weight and prematurity, and high rates of missing data (~25%), only the "at least one risk factor" variable was retained (panel B).

medRxiv preprint doi: https://doi.org/10.1101/2024.09.12.24313545; this version posted September 12, 2024. The copyright holder for this preprint (which was not certified by peer review) is the author/funder, who has granted medRxiv a license to display the preprint in perpetuity.

It is made available under a CC-BY 4.0 International license .

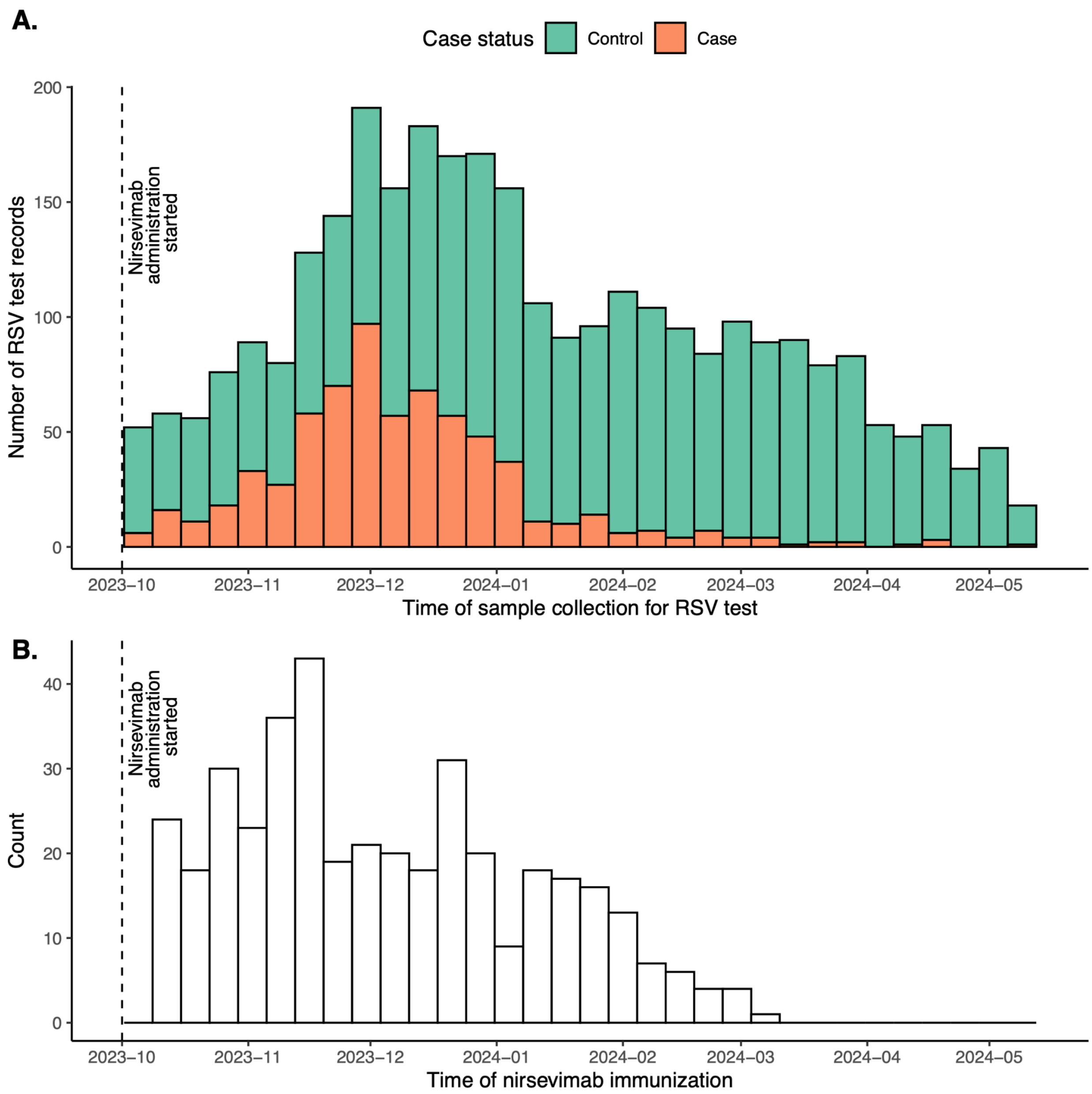

Figure S2. RSV tests and nirsevimab doses during the study period. Panel A shows the number of RSV tests, with orange bars for RSV-positive cases and green bars for RSV-negative controls. Panel B displays the number of nirsevimab doses administered. The vertical dashed line marks the start of nirsevimab administration in Connecticut (October 1, 2023).

medRxiv preprint doi: https://doi.org/10.1101/2024.09.12.24313545; this version posted September 12, 2024. The copyright holder for this preprint (which was not certified by peer review) is the author/funder, who has granted medRxiv a license to display the preprint in perpetuity.

It is made available under a CC-BY 4.0 International license .

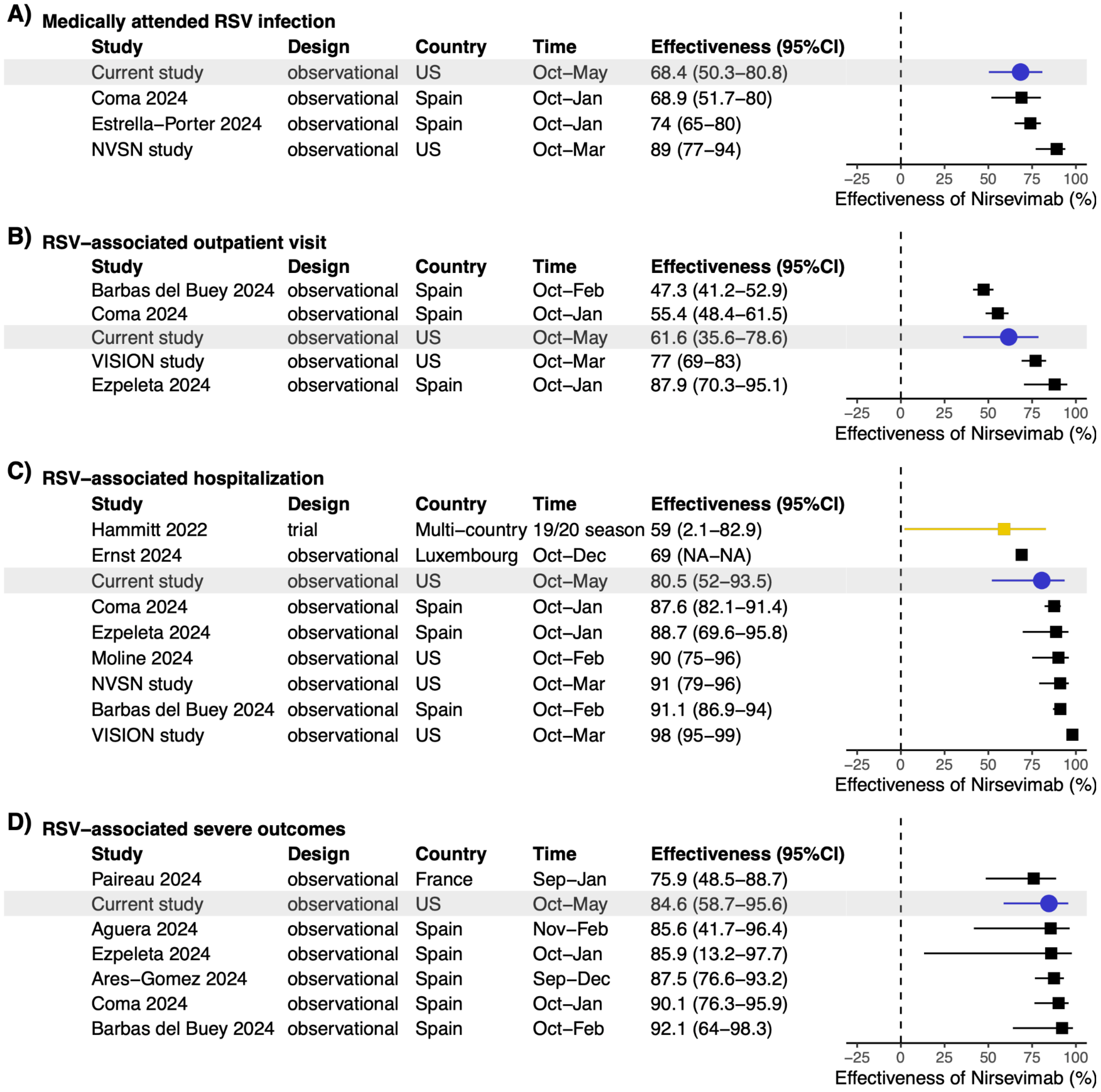

Figure S3. Overview of nirsevimab effectiveness: current study estimates in context with prior research. This figure contrasts adjusted effectiveness (post-licensure) and efficacy (pre-licensure) estimates from prior studies with those from the current study. The right panel shows means (dots) and uncertainty intervals (bars). Gold squares represent Phase IIb/III trial data, black squares represent observational studies, and blue circles represent the current study (highlighted in gray). Ernst et al. 2024[1] did not report uncertainty intervals.

medRxiv preprint doi: https://doi.org/10.1101/2024.09.12.24313545; this version posted September 12, 2024. The copyright holder for this preprint (which was not certified by peer review) is the author/funder, who has granted medRxiv a license to display the preprint in perpetuity.

It is made available under a CC-BY 4.0 International license .

Outcome prevented	RSV positive	RSV negative	Unadjusted Effectiveness (95%CI)	Adjusted Effectiveness (95%CI)
**Medically attended RSV infection**				
Not immunized	659	2101	REF	REF
Immunized (50mg)	15	220	78.3 (64.3–87.7)	66 (42.1–81.4)
Immunized (100mg)	6	89	78.5 (54.7–91.7)	72.7 (40.6–89.6)
Immunized (any dosage)	21	309	78.3 (66.8–86.6)	68.4 (50.3–80.8)
**RSV–associated outpatient visit**				
Not immunized	498	1760	REF	REF
Immunized (50mg)	11	161	75.9 (57.2–87.7)	58.1 (21.5–79.5)
Immunized (100mg)	5	70	74.8 (43.1–91.2)	67.1 (22.9–88.8)
Immunized (any dosage)	16	231	75.5 (60.3–86)	61.6 (35.6–78.6)
**RSV–associated hospitalization**				
Not immunized	161	307	REF	REF
Immunized (50mg)	4	51	85 (62.6–95.5)	77.8 (39.6–93.7)
Immunized (100mg)	1	19	90 (51–99.4)	87.9 (34.3–99.4)
Immunized (any dosage)	5	70	86.4 (68.7–95.3)	80.5 (52–93.5)
**RSV–associated severe outcomes**				
Not immunized	141	212	REF	REF
Immunized (50mg)	3	60	92.5 (79.2–98.2)	84.7 (52.9–96.5)
Immunized (100mg)	1	18	91.6 (58.9–99.5)	85.8 (17.9–99.3)
Immunized (any dosage)	4	78	92.3 (80.9–97.7)	84.6 (58.7–95.6)

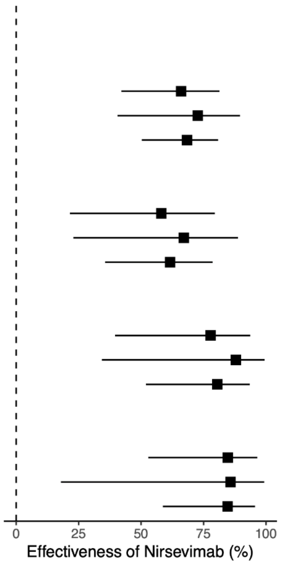

Figure S4. Effectiveness of nirsevimab against RSV infections by dose, clinical setting, and disease severity. Square dots indicate mean effectiveness estimates, with horizontal lines representing 95% confidence intervals. All models adjusted for age and calendar month. Models for RSV-associated hospitalization and severe disease also accounted for the presence of underlying risk factors.

medRxiv preprint doi: https://doi.org/10.1101/2024.09.12.24313545; this version posted September 12, 2024. The copyright holder for this preprint (which was not certified by peer review) is the author/funder, who has granted medRxiv a license to display the preprint in perpetuity.

It is made available under a CC-BY 4.0 International license .

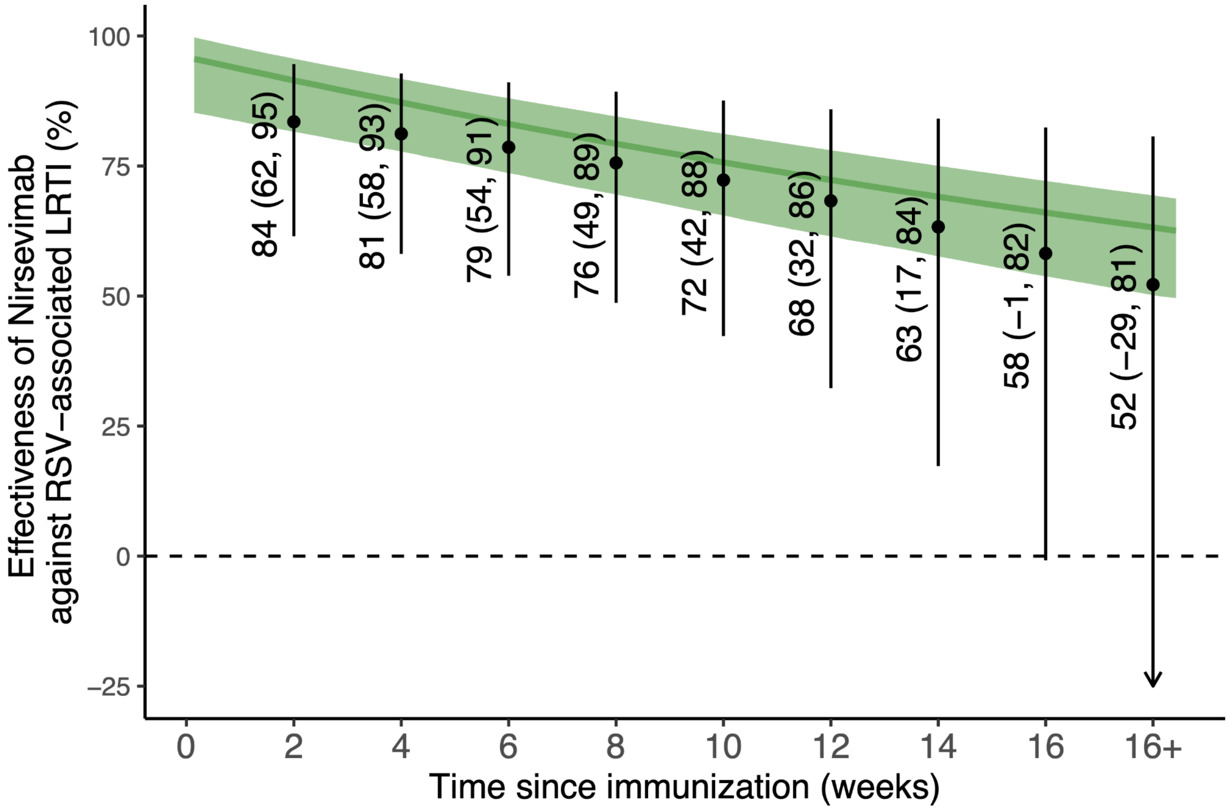

Figure S5. Effectiveness of nirsevimab against RSV-associated LRTI by time since immunization. The green curve and shaded area represent the median and 95% credible interval of the estimated efficacy of nirsevimab reported by Hodgson et al. [2], where efficacy over time was estimated using data from Phase IIb and Phase III trials in a survival model. The black dots denote the median estimates of nirsevimab's effectiveness in preventing RSV-associated LRTI from our current study, using the same endpoint as in Hodgson et al. for comparison [2]. The error bars show the 95% credible intervals for the estimates, and the labels provide the exact values.

medRxiv preprint doi: https://doi.org/10.1101/2024.09.12.24313545; this version posted September 12, 2024. The copyright holder for this preprint (which was not certified by peer review) is the author/funder, who has granted medRxiv a license to display the preprint in perpetuity.

It is made available under a CC-BY 4.0 International license .

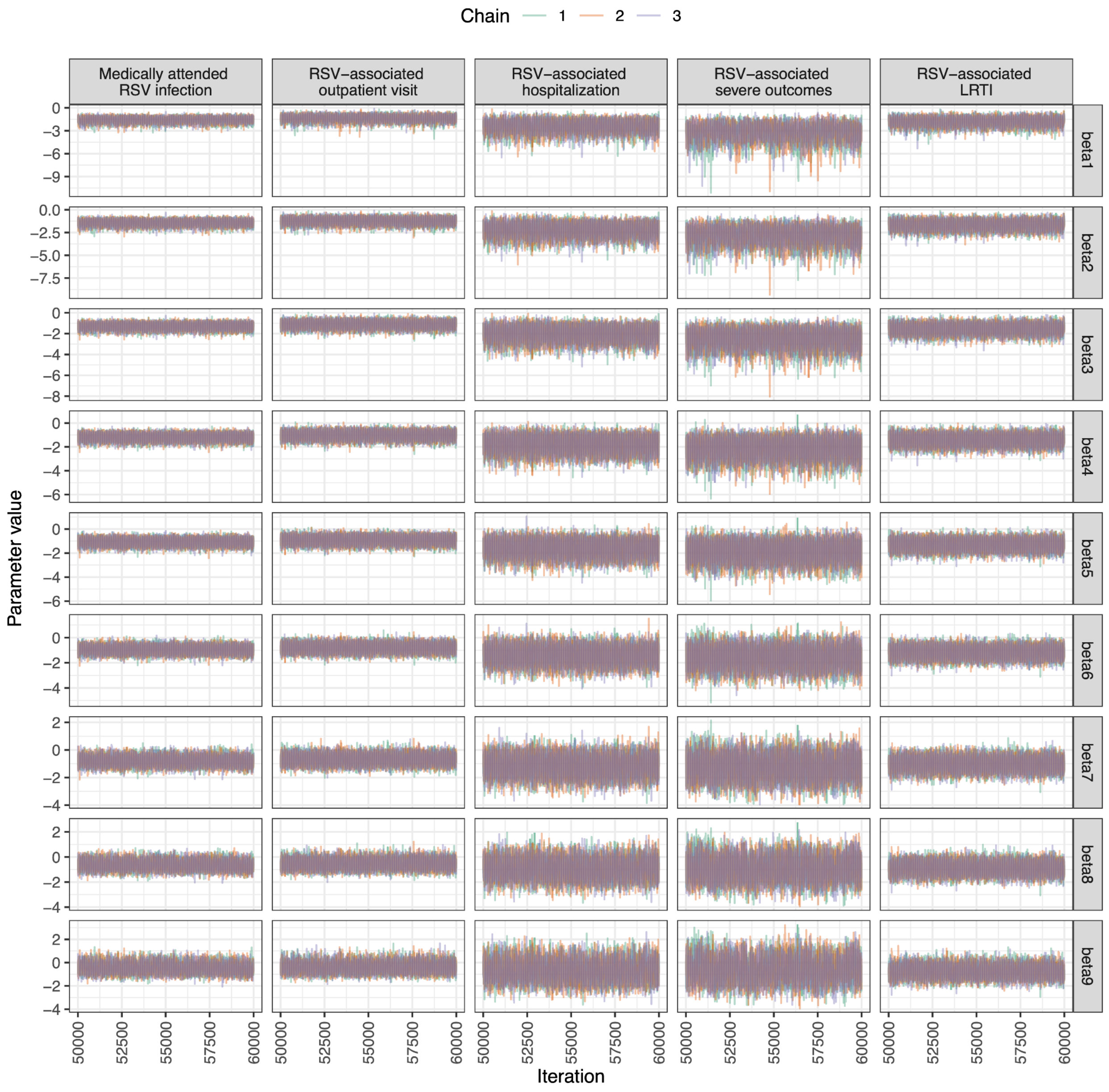

Figure S6. Trace plots for the coefficients of waning effectiveness. Trace plots for β_n (effectiveness coefficient for each biweek interval after nirsevimab immunization, n = 1,2,3,...9) are displayed by the examined outcome. All parameters demonstrate good convergence. Iterations of the burn-in period (iterations 0–50,000) are excluded, and only the sampled iterations (50,000–60,000) are presented.

medRxiv preprint doi: https://doi.org/10.1101/2024.09.12.24313545; this version posted September 12, 2024. The copyright holder for this preprint (which was not certified by peer review) is the author/funder, who has granted medRxiv a license to display the preprint in perpetuity.

It is made available under a CC-BY 4.0 International license .

SECTION 2. SUPPLEMENTAL TABLES REFERENCED IN THE MAIN MANUSCRIPT

Table S1. Definition of key clinical outcomes and risk factors

Acute respiratory illness (ARI) [1]	Risk factors for severe RSV diseases	Medically attended upper respiratory infections (URTI)	Medically attended lower respiratory infections (LRTI)
Acute onset (<10 days) illness that presents with at least two of the following: fever (measured or subjective), chills, rigors, myalgia, headache, sore throat, nausea or vomiting, diarrhea, fatigue, congestion, or one of the following: cough, shortness of breath, difficulty breathing, olfactory disorder, taste disorder, confusion, persistent chest pain, pale, gray, hypoxia, clinical or radiographic evidence of pneumonia or respiratory distress syndrome.	● Prematurity (gestational age less than 37 weeks)<br>● Anemia<br>● Immunodeficiency<br>● Cardiac abnormalities<br>● Pulmonary diseases<br>● Down syndrome<br>● Low birth weight (< 2,500 grams)	● Difficulty breathing<br>● Cough<br>● Croup<br>● Pain in throat / sore throat<br>● Nasal congestion<br>● Acute obstructive laryngitis<br>● Laryngeal stridor<br>● Pharyngitis<br>● Nasopharyngitis<br>● Otitis media<br>● Reactive airway diseases<br>● Upper respiratory tract infections recorded problem list with or without specifying pathogen	● Wheezing<br>● Bronchiolitis<br>● Bronchospasm<br>● Laryngotracheobronchiti s<br>● Acute chest syndrome<br>● Pneumonia<br>● Hypoxia<br>● Hypoxemia<br>● Lower respiratory tract infections recorded in the problem list with or without specifying pathogen

medRxiv preprint doi: https://doi.org/10.1101/2024.09.12.24313545; this version posted September 12, 2024. The copyright holder for this preprint (which was not certified by peer review) is the author/funder, who has granted medRxiv a license to display the preprint in perpetuity.

It is made available under a CC-BY 4.0 International license .

Table S2. Variable selection for the multivariable logistic regression models. The first row for each outcome presents the full model with all potential confounders. In each subsequent row, one variable is removed per step, with the final row showing the confounders included in the final model. Immunization status was included a priori in all models, and the final model was selected based on the lowest Akaike information criterion (AIC) score.

**Outcome**	**Confounders**	**AIC**
Medically attended RSV infection	age_tested + month_tested + race_ethnicity + atleastone_risk_factor + insurance_type	2,853.0
	- race_ethnicity	2,849.0
	- insurance_type	2,846.8
	- atleastone_risk_factor<br>**(Final model:** age_tested + month_tested**)**	2,845.1
RSV-associated outpatient visit	age_tested + month_tested + race_ethnicity + atleastone_risk_factor + insurance_type	2,242.0
	- race_ethnicity	2,238.1
	- insurance_type	2,236.3
	- atleastone_risk_factor<br>**(Final model:** age_tested + month_tested**)**	2,235.6
RSV-associated hospitalization	age_tested + month_tested + race_ethnicity + atleastone_risk_factor + insurance_type	555.2
	- race_ethnicity	552.1
	- insurance_type<br>**(Final model:** age_tested + month_tested + atleastone_risk_factor**)**	550.4
RSV-associated severe outcomes	age_tested + month_tested + race_ethnicity + atleastone_risk_factor + insurance_type	425.4
	**- race_ethnic**ity	419.5
	- insurance_type<br>**(Final model:** age_tested + month_tested + atleastone_risk_factor**)**	419.0
RSV-associated LRTI	age_tested + month_tested + race_ethnicity + atleastone_risk_factor + insurance_type	836.7
	- race_ethnicity	830.7
	- insurance_type<br>**(Final model:** age_tested + month_tested + atleastone_risk_factor**)**	829.4
RSV-associated LRTI hospitalization	age_tested + month_tested + race_ethnicity + atleastone_risk_factor + insurance_type	297.7
	- race_ethnicity	293.9
	- insurance_type<br>**(Final model:** age_tested + month_tested + atleastone_risk_factor**)**	293.0

medRxiv preprint doi: https://doi.org/10.1101/2024.09.12.24313545; this version posted September 12, 2024. The copyright holder for this preprint (which was not certified by peer review) is the author/funder, who has granted medRxiv a license to display the preprint in perpetuity.

It is made available under a CC-BY 4.0 International license .

Table S3. Comparison of immunized and unimmunized patients.

**Characteristic**	**Overall**, N = 3,090[1]	**Immunized**, N = 330[1]	**Unimmunized**, N = 2,760[1]	**Standardized Mean Difference**[1]
**Sex**				0.03
Female	1,317 (42.6%)	138 (41.8%)	1,179 (42.7%)	
Male	1,772 (57.3%)	192 (58.2%)	1,580 (57.2%)	
(Missing)	1 (0.0%)	0 (0.0%)	1 (0.0%)	
**Age at testing (months)**				-0.84
Median (IQR)	6.7 (3.6, 9.7)	3.4 (1.8, 6.0)	7.1 (4.1, 10.0)	
**Race and ethnicity**				0.32
Hispanic	1,328 (43.0%)	138 (41.8%)	1,190 (43.1%)	
White non-Hispanic	820 (26.5%)	68 (20.6%)	752 (27.2%)	
Black non-Hispanic	533 (17.2%)	86 (26.1%)	447 (16.2%)	
Other non-Hispanic[2]	161 (5.2%)	24 (7.3%)	137 (5.0%)	
Unknown	248 (8.0%)	14 (4.2%)	234 (8.5%)	
**Birth weight**				-0.3
Median (IQR)	3,214.3 (2,824.9, 3,563.8)	3,099.6 (2,575.6, 3,483.7)	3,234.4 (2,875.0, 3,573.7)	
(Missing)	783 (25.3%)	26 (7.9%)	757 (27.4%)	
**Gestational age < 37 weeks**	418 (13.5%)	90 (27.3%)	328 (11.9%)	0.63
(Missing)	757 (24.5%)	22 (6.7%)	735 (26.6%)	
**Pulmonary diseases**	156 (5.0%)	19 (5.8%)	137 (5.0%)	0.04
**Cardiac diseases**	152 (4.9%)	33 (10.0%)	119 (4.3%)	0.22
**Anemia**	94 (3.0%)	11 (3.3%)	83 (3.0%)	0.02
**Having at least one risk factor**[3]	750 (24.3%)	123 (37.3%)	627 (22.7%)	0.32
**Insurance type**				0.24
Private	983 (31.8%)	78 (23.6%)	905 (32.8%)	
Public	2,088 (67.6%)	252 (76.4%)	1,836 (66.5%)	
Uninsured	19 (0.6%)	0 (0.0%)	19 (0.7%)	

Data are presented as median (IQR) for continuous measures and n/total (%) for categorical measures.

[1]Standardized mean difference: the difference in means between case and control participants in units of the pooled SD. Covariates with an absolute standardized mean difference greater than 0.2 were considered to have important imbalances.

[2]Inclusing Asian, Pacific Islander, Middle Eastern or Northern American, American Indian, or Native American by self-reporting.

[3]Have at least one of the following conditions recorded in the infant's medical history or diagnosis records: 1) Anemia; 2) Immunodeficiency (e.g. transplantation history, leukemia, etc.); 3) Cardiac diseases (including congenital heart diseases diagnosed at birth or any reporting of heart conditions); 4) Pulmonary diseases; 5) Down syndrome; 6) Small for gestational age (birth weight < 2,500 grams); 7) Prematurity (gestational age less than 37 weeks).

medRxiv preprint doi: https://doi.org/10.1101/2024.09.12.24313545; this version posted September 12, 2024. The copyright holder for this preprint (which was not certified by peer review) is the author/funder, who has granted medRxiv a license to display the preprint in perpetuity.

It is made available under a CC-BY 4.0 International license .

Table S4. Clinical characteristics of RSV-positive cases

Characteristic	**Overall**, N = 680[1]	**Unimmunized**, N = 659	**Immunized**, N = 21[1]
**Hospital admission**	166 (24.4%)	161 (24.4%)	5 (23.8%)
**Duration of hospitalization (days)**			
Median (IQR)	1.0 (1.0, 2.0)	1.0 (1.0, 2.0)	3.0 (2.0, 3.0)
N missing (% missing)	514 (75.6%)	498 (75.6%)	16 (76.2%)
**ICU admission**	23 (3.4%)	22 (3.3%)	1 (4.8%)
**Duration of ICU admission (days)**			
Median (IQR)	3.1 (1.8, 7.3)	3.5 (1.8, 7.4)	2.4 (2.4, 2.4)
N missing (% missing)	657 (96.6%)	637 (96.7%)	20 (95.2%)
**Required high-flow oxygen support**	145 (21.3%)	141 (21.4%)	4 (19.0%)
**Upper respiratory tract infection (URTI)**[2]	229 (33.7%)	221 (33.5%)	8 (38.1%)
**Lower respiratory tract infection (LRTI)**[3]	361 (53.1%)	350 (53.1%)	11 (52.4%)
**Fever (> 38°C/100.4°F)**	239 (35.1%)	235 (35.7%)	4 (19.0%)
**Cough**	98 (14.4%)	94 (14.3%)	4 (19.0%)
**Wheezing**	9 (1.3%)	9 (1.4%)	0 (0.0%)
**Breathing difficulties**	3 (0.4%)	3 (0.5%)	0 (0.0%)
**Bronchiolitis**	349 (51.3%)	338 (51.3%)	11 (52.4%)
**Sepsis**	2 (0.3%)	2 (0.3%)	0 (0.0%)

[1]Data are presented as median (IQR) for continuous measures and n/total (%) for categorical measures.

[2,3] See Table S1 for definitions of URTI and LRTI.

medRxiv preprint doi: https://doi.org/10.1101/2024.09.12.24313545; this version posted September 12, 2024. The copyright holder for this preprint (which was not certified by peer review) is the author/funder, who has granted medRxiv a license to display the preprint in perpetuity.

It is made available under a CC-BY 4.0 International license .

Table S5. Sensitivity Analysis.

	Sensitivity Analysis	Exposure	Cases	Controls	Unadjusted effectiveness (95% CI)	Adjusted effectiveness (95% CI)
**Alternative Exposure Definitions***						
	Hepatitis B vaccine as 'sham' exposure	Immunized	365	1363	11 (-5.6-25)	-7 (-28.8-11)
		Unimmunized	315	1047		
	Immunized ≤ 7 days before testing	Immunized	20	297	78.4 (66.7-86.8)	66.7 (47.2-80)
		Unimmunized	660	2113		
**Alternative Case Definition+**						
	RSV-associated LRTI[1]	Immunized	11	57	82.1 (66.6-91.2)	71.7 (42-87.1)
		Unimmunized	350	325		
	RSV-associated LRTI hospitalization	Immunized	5	22	81.5 (53.3-94)	67.4 (-2.3-90.9)
		Unimmunized	145	118		
**Alternative Control Definition***						
	Only positive for other viruses as controls[2]	Immunized	21	27	79.7 (63.3-88.9)	78.2 (54.8-89.7)
		Unimmunized	659	172		
**Alternative Sample Selection***						
	Exclude high-risk infants[3]	Immunized	21	304	77.7 (65.8-86.2)	68 (49.8-80.6)
		Unimmunized	541	1745		
	Exclude infants with pre-existing immunity[4]	Immunized	17	247	77.3 (63.3-86.9)	66.6 (44.6-81)
		Unimmunized	265	874		

[1] Lower respiratory tract infection (LRTI): see definition in Table S1.
[2] Other viruses: Influenza A, influenza B, adenovirus, rhinovirus, and parainfluenza.
[3] Excluded infants older than 8 months.
[4] Excluded infants whose mother was immunized by RSV maternal vaccine and infants possibly exposed during the last RSV season (born before July 1st, 2023).
*Effectiveness against medically attended RSV infection, adjusted for age tested and calendar month.
+ Effectiveness against RSV-associated LRTI or LRTI hospitalization, adjusted for age tested, calendar month, and having at least one risk factor.

medRxiv preprint doi: https://doi.org/10.1101/2024.09.12.24313545; this version posted September 12, 2024. The copyright holder for this preprint (which was not certified by peer review) is the author/funder, who has granted medRxiv a license to display the preprint in perpetuity.

It is made available under a CC-BY 4.0 International license .

SECTION 3. SUPPLEMENTAL METHODS

Estimating the effectiveness of nirsevimab over time

We evaluated the waning of nirsevimab's protective effect over time using a logistic regression model within a Bayesian framework. For the jth test record in our dataset, the observed case status (i.e., whether the patient tested positive or negative for RSV) followed a Bernoulli distribution, such that

$$Case\_Status_j \sim Bernoulli(p_j).$$

The time between vaccination with nirsevimab and sample collection for RSV testing was categorized into nine bi-weekly intervals (2, 4, 6, ... >16 weeks). To incorporate this variable in the regression model, we created dummy variables to represent each time category ($time\_since\_vax_{jn}, n = 1,2,3 \ldots 9$). For example, if an individual was vaccinated 0-2 weeks before testing, $time\_since\_vax_{j1} = 1$ and $time\_since\_vax_{jn} = 0$ for $n = 2,3, \ldots 9$. For an unvaccinated individual, all the dummy variables $time\_since\_vax_{jn}$ ($n = 1,2,3, \ldots 9$) took the value of 0. The probability of testing positive, p_j, was modeled using a multivariate logistic regression framework as follows:

$$logit(p_j) = \beta_0 + \sum_{n=1}^{N} \beta_n time\_since\_vax_{jn} + \sum_{m=1}^{M} \gamma_m Z_{jm}$$

where Z_{jm} represents potential confounders (e.g., age at testing, month of testing, risk factors), and γ_m is the coefficients for the confounders.

Due to the waning nature of passive immunity, we assumed that nirsevimab's effectiveness had a non-increasing trend over time. To reflect this in the model, we imposed a monotonic structure on the regression coefficients β_n's, such that

$$\beta_{n+1} = \beta_n + d_{n+1}, n = 1, ,3, \ldots 9$$

$$d_{n+1} \sim Normal(0, \sigma_d^2) T(0,)$$

$$\sigma_d^2 \sim Inverse\ Gamma(0.01, 0.01)$$

$T(0,)$ represents truncation at 0, allowing d_{n+1} to take only non-negative values. For β_1 (coefficient for the effectiveness 0-2 weeks after vaccination), we used a weakly informative prior distribution:

$$\beta_1 \sim Normal(0, 100^2)$$

We examined effectiveness over time against various clinical endpoints. The model for each endpoint was fitted separately in the rjags package in R version 4.3.1, in which we collected

medRxiv preprint doi: https://doi.org/10.1101/2024.09.12.24313545; this version posted September 12, 2024. The copyright holder for this preprint (which was not certified by peer review) is the author/funder, who has granted medRxiv a license to display the preprint in perpetuity.

It is made available under a CC-BY 4.0 International license .

10,000 samples from the posterior distribution after discarding the first 50,000 samples in the burn-in period. Convergence was evaluated using trace plots (Figure S6). The estimated effectiveness of nirsevimab after a given period of time (for time interval n) since immunization IE_n was calculated as

$$IE_n = \left(1 - e^{\beta_n}\right) * 100\%, n = 1,2, \dots 9$$

Medians and 95% quantile-based credible intervals were calculated from the collected posterior samples.

medRxiv preprint doi: https://doi.org/10.1101/2024.09.12.24313545; this version posted September 12, 2024. The copyright holder for this preprint (which was not certified by peer review) is the author/funder, who has granted medRxiv a license to display the preprint in perpetuity.

It is made available under a CC-BY 4.0 International license .

STROBE Statement—Checklist of items that should be included in reports of ***case-control studies***

	Item No	Recommendation	Page No
**Title and abstract**	1	(*a*) Indicate the study's design with a commonly used term in the title or the abstract	1,3
		(*b*) Provide in the abstract an informative and balanced summary of what was done and what was found	3
**Introduction**			
Background/rationale	2	Explain the scientific background and rationale for the investigation being reported	4
Objectives	3	State specific objectives, including any prespecified hypotheses	4
**Methods**			
Study design	4	Present key elements of study design early in the paper	4
Setting	5	Describe the setting, locations, and relevant dates, including periods of recruitment, exposure, follow-up, and data collection	4-5
Participants	6	(*a*) Give the eligibility criteria, and the sources and methods of case ascertainment and control selection. Give the rationale for the choice of cases and controls	5
		(*b*) For matched studies, give matching criteria and the number of controls per case	NA
Variables	7	Clearly define all outcomes, exposures, predictors, potential confounders, and effect modifiers. Give diagnostic criteria, if applicable	5
Data sources/ measurement	8*	For each variable of interest, give sources of data and details of methods of assessment (measurement). Describe comparability of assessment methods if there is more than one group	5
Bias	9	Describe any efforts to address potential sources of bias	5-6
Study size	10	Explain how the study size was arrived at	5
Quantitative variables	11	Explain how quantitative variables were handled in the analyses. If applicable, describe which groupings were chosen and why	5
Statistical methods	12	(*a*) Describe all statistical methods, including those used to control for confounding	5-6
		(*b*) Describe any methods used to examine subgroups and interactions	NA
		(*c*) Explain how missing data were addressed	NA
		(*d*) If applicable, explain how matching of cases and controls was addressed	NA
		(*e*) Describe any sensitivity analyses	6
**Results**			
Participants	13*	(a) Report numbers of individuals at each stage of study—eg numbers potentially eligible, examined for eligibility, confirmed eligible, included in the study, completing follow-up, and analysed	7
		(b) Give reasons for non-participation at each stage	NA
		(c) Consider use of a flow diagram	10
Descriptive data	14*	(a) Give characteristics of study participants (eg demographic, clinical, social) and information on exposures and potential confounders	7, 14
		(b) Indicate number of participants with missing data for each variable of interest	14
Outcome data	15*	Report numbers in each exposure category, or summary measures of exposure	14

medRxiv preprint doi: https://doi.org/10.1101/2024.09.12.24313545; this version posted September 12, 2024. The copyright holder for this preprint (which was not certified by peer review) is the author/funder, who has granted medRxiv a license to display the preprint in perpetuity.

It is made available under a CC-BY 4.0 International license .

Main results	16	(*a*) Give unadjusted estimates and, if applicable, confounder-adjusted estimates and their precision (eg, 95% confidence interval). Make clear which confounders were adjusted for and why they were included	7,11
		(*b*) Report category boundaries when continuous variables were categorized	NA
		(*c*) If relevant, consider translating estimates of relative risk into absolute risk for a meaningful time period	NA
Other analyses	17	Report other analyses done—eg analyses of subgroups and interactions, and sensitivity analyses	8
**Discussion**			
Key results	18	Summarise key results with reference to study objectives	8
Limitations	19	Discuss limitations of the study, taking into account sources of potential bias or imprecision. Discuss both direction and magnitude of any potential bias	9
Interpretation	20	Give a cautious overall interpretation of results considering objectives, limitations, multiplicity of analyses, results from similar studies, and other relevant evidence	9
Generalisability	21	Discuss the generalisability (external validity) of the study results	9
**Other information**			
Funding	22	Give the source of funding and the role of the funders for the present study and, if applicable, for the original study on which the present article is based	1

*Give information separately for cases and controls.

Note: An Explanation and Elaboration article discusses each checklist item and gives methodological background and published examples of transparent reporting. The STROBE checklist is best used in conjunction with this article (freely available on the Web sites of PLoS Medicine at http://www.plosmedicine.org/, Annals of Internal Medicine at http://www.annals.org/, and Epidemiology at http://www.epidem.com/). Information on the STROBE Initiative is available at http://www.strobe-statement.org.

medRxiv preprint doi: https://doi.org/10.1101/2024.09.12.24313545; this version posted September 12, 2024. The copyright holder for this preprint (which was not certified by peer review) is the author/funder, who has granted medRxiv a license to display the preprint in perpetuity.

It is made available under a CC-BY 4.0 International license .

SECTION 4. SUPPLEMENTAL REFERENCES

1. Ernst, C., et al., Impact of nirsevimab prophylaxis on paediatric respiratory syncytial virus (RSV)-related hospitalisations during the initial 2023/24 season in Luxembourg. Euro Surveill, 2024. 29(4).
2. Hodgson, D., et al., Protecting infants against RSV disease: an impact and cost-effectiveness comparison of long-acting monoclonal antibodies and maternal vaccination. Lancet Reg Health Eur, 2024. 38: p. 100829.
3. Ezpeleta, G., et al., Effectiveness of nirsevimab immunoprophylaxis administered at birth to prevent infant hospitalisation for respiratory syncytial virus infection: A population-based cohort study. Vaccines, 2024. 12(4): p. 383.
4. Hammitt, L.L., et al., Nirsevimab for Prevention of RSV in Healthy Late-Preterm and Term Infants. N Engl J Med, 2022. 386(9): p. 837-846.
5. Summary of effectiveness of nirsevimab in infants. 2024; Available from: https://www.cdc.gov/vaccines/acip/meetings/downloads/slides-2024-06-26-28/04-RSV-Mat-Peds-Payne-508.pdf.
6. Estrella-Porter, P., et al., Effectiveness of nirsevimab introduction against respiratory syncytial virus in the Valencian Community: A preliminary assessment. Vaccine, 2024. 42(22): p. 126030.
7. Coma, E., et al., Effectiveness of nirsevimab immunoprophylaxis against respiratory syncytial virus-related outcomes in hospital and primary care settings: a retrospective cohort study in infants in Catalonia (Spain). Arch Dis Child, 2024. 109(9): p. 736-741.
8. Barbas Del Buey, J.F., et al., The effectiveness of nirsevimab in reducing the burden of disease due to respiratory syncytial virus (RSV) infection over time in the Madrid region (Spain): a prospective population-based cohort study. Frontiers in Public Health, 2024. 12: p. 1441786.
9. Moline, H.L., Early Estimate of Nirsevimab Effectiveness for Prevention of Respiratory Syncytial Virus–Associated Hospitalization Among Infants Entering Their First Respiratory Syncytial Virus Season—New Vaccine Surveillance Network, October 2023–February 2024. MMWR. Morbidity and Mortality Weekly Report, 2024. 73.
10. Paireau, J., et al., Nirsevimab Effectiveness Against Cases of Respiratory Syncytial Virus Bronchiolitis Hospitalised in Paediatric Intensive Care Units in France, September 2023-January 2024. Influenza Other Respir Viruses, 2024. 18(6): p. e13311.
11. Agüera, M., et al., Nirsevimab immunization's real-world effectiveness in preventing severe bronchiolitis: A test-negative case-control study. Pediatr Allergy Immunol, 2024. 35(6): p. e14175.
12. Ares-Gómez, S., et al., Effectiveness and impact of universal prophylaxis with nirsevimab in infants against hospitalisation for respiratory syncytial virus in Galicia, Spain: initial results of a population-based longitudinal study. The Lancet Infectious Diseases, 2024.
